# When Your Body Tells You to Not Breastfeed—The Connivance of Oxytocin, Prolactin, and Dopamine

**DOI:** 10.3390/ijms26125909

**Published:** 2025-06-19

**Authors:** Vladimír Kraus, Beáta Čižmárová, Anna Birková

**Affiliations:** 1 Department of Gyneacology and Obstetrics, Faculty of Medicine, Pavol Jozef Šafárik University in Košice, Trieda SNP 1, 040 11 Košice, Slovakia; vladimir.kraus1@upjs.sk; 2 Department of Medical and Clinical Biochemistry, Faculty of Medicine, Pavol Jozef Šafárik University in Košice, Trieda SNP 1, 040 11 Košice, Slovakia; anna.birkova@upjs.sk

**Keywords:** oxytocin, prolactin, dopamine, dysphoric milk ejection reflex, D-MER, breastfeeding aversion/agitation, BAA, breastfeeding aversion response

## Abstract

Breastfeeding is universally recognized for its extensive health benefits for both infants and mothers. However, for some women, the experience of breastfeeding can be complicated by intense negative emotional and physical reactions, including phenomena such as dysphoric milk ejection reflex and breastfeeding aversion/agitation. This review explores the neuroendocrine underpinnings of these conditions, emphasizing the interplay between oxytocin, prolactin, and dopamine. Oxytocin, traditionally viewed as a hormone promoting bonding and emotional regulation, can paradoxically provoke a stress response in vulnerable individuals. Prolactin, a key hormone for lactation and maternal behaviors, is implicated in stress resilience and mood regulation, but its dysregulation may contribute to depressive states. Dopamine, critical for reward processing and emotional stability, may underlie the acute emotional dysregulation seen in dysphoric milk ejection reflex. Together, disturbances in these neurohormonal systems may explain the aversive emotional experiences during breastfeeding. An improved understanding of these mechanisms offers critical insights into maternal mental health during lactation and underscores the importance of supportive clinical approaches for affected women.

## 1. Introduction

Breastfeeding, the process of nourishing an infant with milk directly from the mother’s breast, has long been regarded as the optimal method of infant feeding across human history. Global health policies, guided by the principles of the Innocenti Declaration, have consistently prioritized the promotion and support of breastfeeding to improve its prevalence worldwide [1]. The World Health Organization advocates exclusive breastfeeding during the first six months of life to ensure optimal growth, development, and overall health. Following this period, continued breastfeeding alongside the introduction of appropriate complementary foods is recommended for up to two years or more. This guidance is similarly endorsed by the American Academy of Pediatrics [1].

Breast milk represents the optimal source of nutrition for infants, offering a comprehensive array of essential nutrients—including vitamins, minerals, proteins, fats, and carbohydrates—specifically tailored to support their growth and developmental requirements [2]. Often referred to as “liquid gold” due to its complex and beneficial composition, breast milk contains key long-chain polyunsaturated fatty acids such as docosahexaenoic acid and arachidonic acid, which are vital for optimal cognitive development and visual acuity [3]. Moreover, its dynamic composition continuously adapts to meet the evolving needs of the growing infant [4]. Breast milk also offers significant immunological advantages that are essential for the development of a robust immune system in infants. It facilitates the transfer of passive immunity from mother to child through components such as antibodies and white blood cells, which play a critical role in defending against infections. Additionally, breast milk is rich in bioactive molecules—including cytokines, enzymes, and growth factors—that contribute to immune system modulation [5]. The practice of breastfeeding, particularly with skin-to-skin contact, also promotes the transmission of beneficial maternal microbiota, supporting the establishment of a healthy gut microbiome, a key factor in immune maturation [6]. Consequently, breastfed infants exhibit a reduced incidence of respiratory infections, gastrointestinal disorders, and allergic conditions compared to those who are formula-fed [4]. Breastfeeding is associated with a significantly reduced risk of a wide range of illnesses in infants. Breastfed children exhibit lower incidence rates of asthma, obesity, gastrointestinal tract infections, necrotizing enterocolitis, type 2 diabetes, otitis media, respiratory infections, and sudden infant death syndrome. More specifically, breastfeeding is linked to a decreased risk of atopic dermatitis and gastroenteritis, with breastfed infants experiencing fewer episodes of these conditions compared to their formula-fed counterparts [7,8]. Breastfeeding has been associated with several potential long-term benefits extending into adulthood. Individuals who were breastfed as infants may exhibit slightly lower levels of blood pressure and cholesterol compared to those who were never breastfed. Furthermore, breastfeeding may positively influence social-emotional development and enhance the mother-child bond. Notably, evidence suggests a link between breastfeeding and higher IQ scores later in life. A meta-analysis demonstrated that breastfed children tend to have higher IQs than non-breastfed peers, even after adjusting for maternal IQ. Additionally, the combined effects of breastfeeding and active maternal bonding may offer protective benefits against internalizing behavioral problems in children [8,9]. In addition to its biochemical benefits, the maternal-infant interaction during breastfeeding—characterized by physical closeness, eye contact, and skin-to-skin contact—further enhances cognitive and emotional development, fostering secure attachment and early social-emotional skills [4]. Breastfeeding offers several important health benefits for mothers as well. It is associated with a reduced risk of developing certain cancers, particularly breast and ovarian cancer, likely due to hormonal changes during lactation that lower lifetime estrogen exposure. Additionally, breastfeeding contributes to a decreased risk of cardiometabolic diseases, including type 2 diabetes mellitus, hypertension, and cardiovascular disease. Among women with a history of gestational diabetes, breastfeeding has been shown to enhance pancreatic β-cell function, and a systematic review supports its role in lowering the risk of type 2 diabetes [8]. Furthermore, breastfeeding has been linked to a lower risk of postpartum depression (PPD), with evidence indicating a 14% reduction in risk. This protective effect may be attributed to the hormonal and psychological benefits of breastfeeding, including the release of prolactin and oxytocin, which possess antidepressant and anxiolytic properties [10]. Breastfeeding also helps modulate stress by reducing cortisol levels, particularly when combined with skin-to-skin contact. As a result, breastfeeding mothers often report lower levels of perceived stress and depressive symptoms compared to non-breastfeeding mothers [10,11]. Breastfeeding is closely associated with enhanced maternal physical and psychological well-being during the postpartum period. Mothers who breastfeed report fewer physician visits, reduced incidence of gastrointestinal and upper respiratory infections, and fewer psychological and cardiovascular symptoms compared to those who do not breastfeed. The act of providing nourishment directly from the body often instills a sense of accomplishment, increasing self-esteem and maternal confidence. Overcoming the challenges of breastfeeding can further reinforce a mother’s sense of self-assuredness and empowerment [11]. Additionally, breastfeeding fosters a strong emotional bond between mother and infant through the release of oxytocin and the experience of skin-to-skin contact, promoting attachment, love, and trust. This close interaction also encourages a responsive parenting style, as mothers become more attuned to their babies’ cues and needs [4]. The calming effects of oxytocin contribute to maternal stress reduction by counteracting stress hormones. Moreover, breastfeeding can enhance body positivity by empowering women to appreciate their bodies’ capacity to nourish their newborns, supporting self-acceptance in the postpartum period. Finally, breastfeeding often facilitates peer support and the development of a sense of community among mothers, offering emotional and social reinforcement during early motherhood [4,12].

It is critically important to investigate any factors that can disturb or prematurely end breastfeeding due to the wide-ranging implications for both maternal and infant health. Despite the benefits listed earlier, breastfeeding rates often fall short of public health goals, with many mothers discontinuing earlier than planned. Factors that contribute to premature weaning—whether physiological, psychological, social, or systemic—must be thoroughly understood to develop effective support strategies. Emotional or neurobiological disturbances, such as those observed in newly recognized conditions like Dysphoric Milk Ejection Reflex (D-MER) and Breastfeeding Aversion and Agitation (BAA), can profoundly impact a mother’s breastfeeding experience. When these challenges are not recognized or addressed, they can lead to unnecessary cessation, compounded by feelings of guilt, failure, and psychological distress. Understanding and addressing breastfeeding disruptors is therefore essential not only to support optimal nutrition and health outcomes for the infant but also to promote maternal mental health and agency. Comprehensive research into these factors can inform clinical guidelines, enhance lactation support services, and guide public health policies aimed at improving breastfeeding initiation and duration rates. The paucity of clinical research and evidence-based guidelines for D-MER and BAA/BAR means that affected individuals are frequently misdiagnosed, dismissed, or left without adequate support. This review of the two new conditions aims to help in investigating these phenomena, enhance professional education, and reduce the stigma surrounding maternal emotional responses to breastfeeding.

## 2. Why Mothers Stop Breastfeeding Prematurely

Concerns about insufficient milk supply represent one of the primary reasons why women discontinue breastfeeding. In a national survey conducted in the United Kingdom, this was identified as the most frequently reported cause of early weaning [8]. Many mothers perceive that their breast milk is inadequate or that their infant is not sufficiently satiated, a perception that can be influenced by maternal mental health conditions, including eating disorders, as well as anxieties surrounding inadequate nutritional intake [9]. Additionally, delayed lactogenesis may exacerbate worries about milk production. Another commonly cited reason for breastfeeding cessation is an inadequate latch, which can hinder effective milk transfer and often results in maternal discomfort or pain. Painful nipples or breasts pose a significant barrier to sustained breastfeeding and are a leading cause of early weaning [1]. Nipple pain may stem from improper infant positioning and latch, suck disorganization, ankyloglossia (tongue-tie), infant biting, or trauma associated with breast pump use. Sore nipples remain among the most commonly reported factors contributing to the premature discontinuation of breastfeeding [8]. Returning to work is a significant societal factor contributing to the early cessation of breastfeeding. Women who are employed outside the home often breastfeed for a shorter duration compared to those who are not. In regions where paid maternity leave is limited or unavailable—such as the United States—mothers may be compelled to resume work soon after childbirth, thereby reducing the opportunity for sustained direct breastfeeding. Furthermore, workplace-related barriers, including inadequate time, lack of private spaces for milk expression, and insufficient employer support, can hinder a mother’s ability to continue breastfeeding upon returning to work [7]. Several maternal, infant, and contextual factors have been identified as barriers to successful and sustained breastfeeding. The mode of delivery, particularly cesarean section, has been associated with lower rates of exclusive breastfeeding, as women who undergo surgical births are less likely to initiate or maintain breastfeeding. A lack of support from healthcare professionals, family members, and partners can further hinder a mother’s ability to overcome breastfeeding challenges, with feelings of pressure to continue without adequate guidance often contributing to early cessation. Concerns about the safety of medications during lactation may also lead some women to forgo breastfeeding altogether [13]. Unrealistic expectations about the breastfeeding experience can result in disappointment and early discontinuation, especially when mothers are not adequately prepared for potential challenges during the antenatal period [9]. Infant-related issues, such as uncoordinated suck and swallow reflexes in near-term infants, can complicate effective feeding. Maternal obesity has also been linked to difficulties with breastfeeding initiation and duration, potentially due to mechanical challenges with positioning and latching, as well as delayed onset of lactogenesis [1]. Additional factors influencing breastfeeding outcomes include smoking during pregnancy, which is associated with an increased risk of early weaning, and complications during childbirth, which may impede breastfeeding initiation and continuation [14]. The early introduction of formula supplementation without medical necessity can disrupt the establishment of an adequate milk supply, while the early use of pacifiers has similarly been associated with shortened breastfeeding duration [1]. Maternal mental health challenges, particularly PPD and anxiety, are strongly associated with the earlier cessation of breastfeeding. Depressive symptoms experienced during pregnancy or the postpartum period have been shown to negatively influence the duration of breastfeeding [15]. Conversely, some evidence suggests that negative breastfeeding experiences may precede and contribute to the development of depressive symptoms. Maternal anxiety has also been linked to reduced breastfeeding intensity and shorter duration. In general, breastfeeding difficulties are correlated with a higher incidence of depressive symptoms and an increased risk of postpartum depression, both of which may lead to early discontinuation. Furthermore, negative attitudes toward breastfeeding and low maternal confidence in one’s ability to breastfeed are associated with more pronounced depressive symptoms and a greater likelihood of early weaning [16]. The most common reasons for premature stopping of nursing are summarized in Table 1.

## 3. Maternal Mental Status and Breastfeeding

Maternal mental status affects breastfeeding and vice versa. Maternal mental health plays a critical role in shaping breastfeeding initiation, duration, and overall experience. Conversely, breastfeeding itself may typically exert a protective effect against postpartum depression (PPD) through hormonal, physiological, and emotional mechanisms. It has been associated with improved maternal mood, enhanced self-esteem, and strengthened bonding between mother and infant [16]. The relationship between breastfeeding and maternal mental status is likely bidirectional. PPD has been shown to predict a reduced likelihood of sustained exclusive breastfeeding, while exclusive breastfeeding is associated with decreased odds of developing PPD at a later stage. The breastfeeding experience itself—including factors such as pain, latching difficulties, maternal emotional responses, and infant engagement—can function either as a stressor or a buffer for maternal mood, depending on the context. Moreover, the interplay between oxytocin and other key hormones involved in lactation and stress regulation may operate differently in mothers experiencing depressive symptoms, potentially influencing both the emotional experience of breastfeeding and its physiological effects [17]. Depression and anxiety during pregnancy are significant predictors of breastfeeding outcomes. Prenatal depression and elevated pregnancy-related anxiety are associated with a lower intention to breastfeed and the reduced likelihood of initiating breastfeeding. Depressive symptoms during pregnancy are consistently linked to a decreased probability of sustaining exclusive breastfeeding for an extended period, as well as to shorter overall breastfeeding duration. Moreover, antenatal depression is predictive of increased breastfeeding difficulties, further hindering successful lactation. Studies have also found that antenatal depression symptoms are correlated with shorter durations of both any breastfeeding and exclusive breastfeeding. Similarly, antenatal anxiety has been associated with decreased breastfeeding intensity and duration. A positive screening for antenatal depression is strongly associated with lower rates of breastfeeding initiation and continuation [16,17]. In this context, two relatively new disorders are being recognized and talked about in the recent literature—D-MER and BAA. Both of these distinct phenomena can present as negative emotional experiences during breastfeeding. While they are considered physiological in origin and distinct from perinatal mood disorders, they differ in their characteristics.

### 3.1. Dysphoric Milk Ejection Reflex

D-MER is a recently identified and still under-researched condition affecting lactating individuals. The phenomenon was first recognized in 2007 by lactation consultant Alia Macrina Heise, who initially encountered it through her own experience following the birth of her third child [18]. Unlike her previous postpartum periods, Heise reported experiencing sudden and intense dysphoria coinciding specifically with the milk ejection reflex, which she initially misattributed to postpartum depression. Upon recognizing the temporal association between these negative affective episodes and milk letdown (“only when her milk was about to release”), and finding no existing clinical explanations, she gave the condition a descriptive name and launched the website D-MER.org in June 2008 to raise awareness and share information [19]. In collaboration with Diane Wiessinger, Heise later co-authored one of the earliest academic descriptions of D-MER in 2011 [19]. Despite this initial contribution to the literature, empirical studies on D-MER remain sparse, and the condition continues to be frequently overlooked or unrecognized in clinical practice. As was mentioned earlier, D-MER is characterized by the sudden onset of negative emotions—such as sadness, anxiety, or anger—that occur immediately before and during the milk ejection reflex. These emotional responses are typically brief, lasting anywhere from 30 s to 10 min, with most episodes falling within the 1 to 5 min range, although some women report durations shorter than a minute or longer than five minutes [20]. The intensity of these emotions can vary considerably, from a mild, hollow sensation to more severe experiences, including intense rage or even suicidal ideation. One account described the sensation as a visceral reaction in the pit of the stomach, similar to the feeling experienced upon receiving distressing news, though it subsides within a few minutes [21]. D-MER is believed to result from hormonal and neurochemical changes during lactation, particularly a rapid drop in dopamine that occurs in conjunction with the milk ejection reflex, or possibly due to oxytocin release [19]. Despite being a distressing emotional experience, D-MER is distinct from PPD, and the two conditions can coexist. A key distinguishing feature is the acute and short-lived emotional response that is specifically tied to milk let-down, unlike the more persistent mood disturbances characteristic of PPD [22]. While some women report concurrent physical sensations such as nausea or the let-down reflex, these are not considered core features of D-MER. The symptoms of D-MER may begin to subside by approximately three months postpartum, though in some cases, they persist throughout the entire duration of lactation [18]. One of the earliest studies on D-MER, a retrospective chart review conducted at a U.S. military medical center, reported an incidence of 9.1% (n = 164) among mothers between 6 and 8 weeks postpartum [21]. Subsequent research has indicated potentially higher prevalence rates. A study conducted in New York reported that 26.9% of 78 new mothers experienced D-MER [23]. In Germany, a larger-scale study involving 1469 lactating parents found a prevalence rate of 14.2% [24]. In Japan, a self-administered survey targeting mothers of three-year-old children revealed that 23.3% (47 out of 202) reported experiencing D-MER with at least one child during breastfeeding [25]. D-MER can significantly impact a mother’s ability and motivation to continue breastfeeding, often leading to early weaning or consideration of cessation well before the intended duration. The sudden and recurrent onset of negative emotions associated with D-MER can undermine a woman’s breastfeeding experience, reducing her desire to persist despite her original intentions. Empirical data highlight the extent of this impact. In one survey, 35.4% of respondents reported that they had either already discontinued breastfeeding or were contemplating doing so due to their D-MER symptoms [21]. Similarly, a separate study found that 16.9% of mothers with D-MER had stopped breastfeeding because of the condition, while an additional 19.2% had considered stopping [24]. This brings the total proportion of mothers affected to approximately 36.1%, closely aligning with the 35% figure reported in the earlier survey. These findings underscore the disruptive influence of D-MER on breastfeeding continuation and highlight the need for increased awareness, screening, and support to help affected mothers manage their symptoms and achieve their breastfeeding goals.

#### 3.1.1. Symptomatology of D-MER

**Figure 1:**
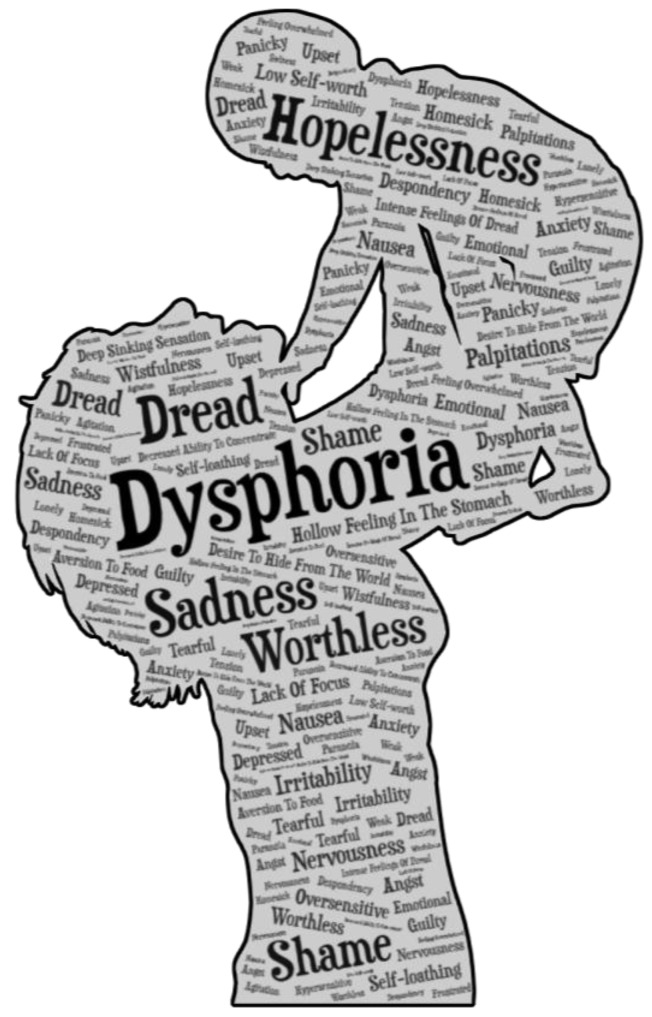
Cornucopia of emotions associated with D-MER.

D-MER is characterized by sudden, brief waves of negative emotions that occur just before and during milk ejection, typically resolving within seconds to a few minutes. The emotional intensity of D-MER can vary significantly (as depicted by Figure 1), ranging from mild discomfort to severe psychological distress. Affected mothers report a spectrum of negative emotional experiences, which can be grouped into several categories. In terms of despondency or dejection, women may describe feelings of hopelessness, sadness, a hollow sensation in the stomach, despair, wistfulness, homesickness, apprehension, or a sense of being emotionally drained. Anxiety-related symptoms may include nervousness, dread, panic, a sense of being overwhelmed, oversensitivity, or an intense fear of impending doom. Irritability and anger are also commonly reported, manifesting as tension, agitation, impatience, irritability, or even paranoia.

In more severe cases, D-MER may be accompanied by intense self-loathing and, in rare instances, suicidal ideation. These symptoms are transient and are uniquely triggered by the physiological process of milk let-down, distinguishing D-MER from other mood disorders such as PPD or BAA [20,26]. The onset of symptoms typically occurs within the first few days postpartum, coinciding with the onset of copious milk production known as lactogenesis II. For many women, symptoms begin 2 to 3 days after birth, although they can emerge anytime within the first week of breastfeeding or pumping. Rare occurrence was a report about the onset of symptoms after 1 month postpartum, with specific cases occurring at 3 months, 1 year, and even 2 years postpartum [25]. The occurrence of symptoms can vary widely among individuals. For many women, symptoms tend to lessen or resolve by approximately three months postpartum, coinciding with the stabilization of lactation and hormonal regulation. However, in some cases, D-MER persists for a longer duration, occasionally continuing throughout the entire breastfeeding period until weaning. One study reported that a substantial proportion of women experienced D-MER symptoms lasting from six months to over a year, indicating that while the condition may be transient for some, it can represent a prolonged challenge for others [21].

#### 3.1.2. Pathophysiology of D-MER

**Figure 2:**
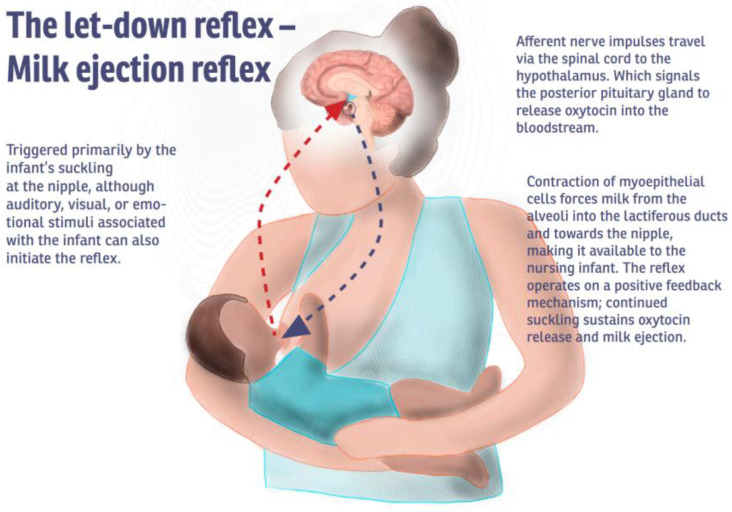
Illustration of the physiological “let down” reflex, also known as milk ejection reflex.

The milk ejection reflex, commonly referred to as the “let-down reflex” is a neuroendocrine mechanism essential for the initiation and continuation of breastfeeding (Figure 2). Triggered by stimulation of the nipple and areola, through infant suckling, it activates sensory pathways via the fourth intercostal nerve and transmits signals to the hypothalamus. In response, the hypothalamus signals the posterior pituitary gland to release oxytocin, a hormone that induces the contraction of myoepithelial cells surrounding the alveoli in the mammary glands. This contraction propels milk from the alveoli into the lactiferous ducts, making it accessible to the nursing infant [27].

Multiple milk ejection reflexes can occur within a single breastfeeding or milk expression session. While some women are acutely aware of the reflex and report sensations such as a warm tingling feeling in the breasts, others may not consciously perceive it [18]. The efficiency and reliability of the reflex can be influenced by various psychological and physiological factors. Positive stimuli—such as hearing the infant cry, thinking about the baby, or anticipating a feeding—can enhance oxytocin release and facilitate the milk let down. Conversely, maternal stress, anxiety, fear, or physical pain can inhibit the reflex by interfering with oxytocin secretion [20]. The milk ejection reflex is also linked to changes in other hormonal pathways. Specifically, during the milk ejection reflex activation, the hypothalamic release of dopamine is suppressed, which allows for an increase in prolactin levels. Prolactin is the primary hormone responsible for milk synthesis and is critical for maintaining milk supply. Consequently, the let-down reflex is not only vital for milk transfer but also supports continued lactation [18].

At this moment, D-MER is thought to be a neuroendocrine disorder with behavioral symptoms. There are two main theories, trying to explain it.

**Figure 3:**
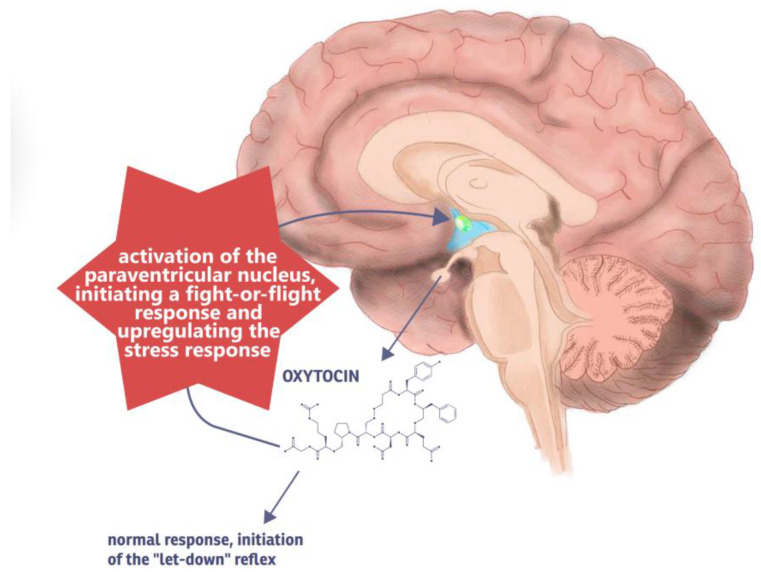
D-MER/oxytocin theory: aberrant signaling of oxytocin responsible for negative sensations.

The oxytocin theory offers a compelling explanation for the physiological underpinnings of D-MER, suggesting that the hormone oxytocin—normally associated with positive parental emotional states—paradoxically triggers a stress response in some women rather than its typical calming effects [28], as illustrated by Figure 3. Under typical conditions, oxytocin facilitates milk ejection and is linked to feelings of well-being, bonding, and affiliation. It also contributes to the downregulation of neuroendocrine stress signaling and has been shown to alleviate symptoms of anxiety and depression. In most individuals, the upregulation of oxytocin during breastfeeding suppresses the stress response, fostering a sense of calm and connection [28]. However, in women with D-MER, particularly those with a history of adversity—such as post-traumatic stress disorder (PTSD), a stressful pregnancy or labor experience, or exposure to synthetic oxytocin during childbirth—the expected response may be altered. It is hypothesized that in these individuals, activation of the paraventricular nucleus of the hypothalamus may instead initiate a fight-or-flight response, effectively reversing the usual emotional effects of oxytocin. In this altered state, oxytocin appears to upregulate rather than downregulate the stress response, leading to the dysphoric emotions characteristic of D-MER [28].

Some researchers propose that this phenomenon may result from a “miswiring” of oxytocin pathways in the brain, causing the milk ejection reflex to be associated with negative emotional responses rather than positive reinforcement. Drawing parallels with PTSD, they suggest that treatment approaches for D-MER might involve efforts to “rewire” these neuroendocrine circuits. Strategies aimed at enhancing oxytocin’s calming effects—such as skin-to-skin contact, mindfulness, and relaxation techniques—are recommended to promote a shift toward a more typical oxytocin response and reduce stress-related symptoms during breastfeeding [20,28].

**Figure 4:**
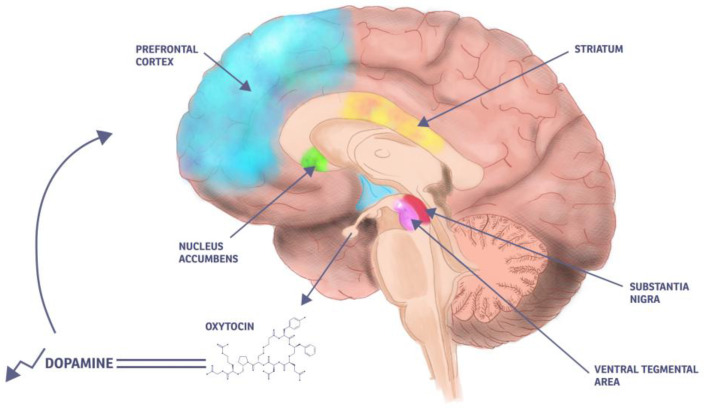
D-MER/dopamine theory: drop of dopamine and its areas of effect.

The dopamine theory presents an alternative neurochemical explanation for the onset of D-MER, proposing that the condition is triggered by a brief but abnormal drop in dopamine levels that occurs immediately prior to the milk ejection reflex MER. Dopamine is a key neurotransmitter associated with mood regulation, motivation, and reward. It plays a crucial role in maintaining emotional stability and a sense of well-being. During lactation, dopamine also functions as an inhibitor of prolactin, the hormone essential for milk production. When the milk ejection reflex is initiated, the release of oxytocin is accompanied by a decrease in dopamine secretion from the hypothalamus, thereby allowing prolactin levels to rise and support continued lactation [20]. According to this theory, in women with D-MER, the typical decrease in dopamine may be either more abrupt or more substantial than normal, resulting in a transient but significant dopamine deficiency. This sudden drop may trigger the acute onset of negative emotional states—such as sadness, anxiety, or irritability—that characterize D-MER [18], as illustrated by Figure 4.

Support for the dopamine theory comes from anecdotal reports indicating that interventions known to elevate or stabilize dopamine levels can alleviate or even eliminate D-MER symptoms in some individuals. For example, pseudoephedrine, a medication known to suppress prolactin by increasing dopamine, was reported to completely resolve D-MER in a case study. Similarly, dopamine reuptake inhibitors such as bupropion have shown potential in reducing D-MER symptoms [29]. However, this theory is not without controversy. Some researchers question the central role of dopamine in D-MER, arguing that the extremely rapid onset and resolution of symptoms more closely align with oxytocin activity. They also contend that dopamine’s primary regulatory function is on prolactin, not oxytocin, suggesting that the oxytocin pathway may offer a more plausible explanation for the acute emotional shifts observed in D-MER [28].

### 3.2. Breastfeeding Aversion/Agitation

The phenomenon of BAA was first described in nonacademic breastfeeding literature in 2003. This early recognition prompted the formation of online support communities, such as www.breastfeedingaversion.com, which served as platforms for sharing personal experiences and gathering anecdotal data. Over time, insights from these communities were incorporated into subsequent editions of international breastfeeding resources. Despite this growing informal awareness, empirical research and peer-reviewed publications on BAA remained limited for many years. Interest in the condition began to increase more noticeably from 2017 onward, with gradual advances in clinical inquiry and recognition within lactation and maternal health fields [30]. BAA is not yet formally classified as a medical or psychiatric condition, nor is it included in standard diagnostic manuals. Much of what is known comes from qualitative reports, surveys, and personal narratives, which are gaining traction among lactation consultants, midwives, and mental health professionals.

BAA, also known as breastfeeding aversion response (BAR), is a phenomenon characterized by the onset of negative emotions and physical discomfort that some women experience specifically while their infant is latched and actively breastfeeding. These aversive responses are distinct from feelings of regret or sadness associated with not breastfeeding or with early weaning, as BAA occurs during the act of breastfeeding itself, despite a mother’s desire or intention to continue nursing [31]. BAA is marked by a range of distressing emotional and physical sensations that occur while the infant is latched and nursing. Mothers experiencing BAA/BAR commonly report feelings of anger, rage, agitation, and irritability, often accompanied by physical discomfort such as a skin-crawling or itchy sensation. Many describe a compelling urge to unlatch the infant or to physically remove them from the breast. In more severe cases, these reactions may escalate to intense feelings of repulsion, disgust, and an aversion to being touched. Some women have described a visceral, overwhelming emotional response, including impulses to scream, hit objects, or, in extreme cases, intrusive thoughts of harming their baby. These experiences are often described as deeply unsettling and are accompanied by a powerful desire to distance oneself from the infant during feeding [20,30]. The negative sensations associated with BAA typically persist for the duration of the breastfeeding session. These distressing emotions and physical discomforts are often continuous while the infant remains latched. However, for many women, the aversive feelings subside almost immediately once the breastfeeding session concludes, providing temporary relief until the next feeding [30]. Although the precise cause of BAA remains unclear, several potential triggers have been identified through anecdotal reports and emerging research. Common triggers include tandem feeding (breastfeeding two children), breastfeeding during pregnancy, and breastfeeding a first child. Hormonal fluctuations, particularly those associated with the menstrual cycle, have also been implicated, with some women experiencing BAA in the days leading up to their period or in relation to monthly hormonal changes [30,32]. Additional reported triggers involve the child’s physical behavior during feeding, such as fidgeting, wriggling, or nipple tweaking, which can intensify maternal discomfort. Maternal fatigue, sleep deprivation, and the feeling of being “touched out” from constant physical contact are also frequently cited as contributing factors. These physical and emotional stressors may amplify the aversive response experienced during breastfeeding sessions [31]. The emotional impact of BAA can be profound, often leaving mothers grappling with intense guilt and shame. This emotional distress typically arises from the internal conflict between experiencing negative, aversive feelings during breastfeeding and the deep desire to nurture and bond with their child. Many mothers report feeling confused or distressed by these emotions, particularly if they previously found breastfeeding to be a positive and fulfilling experience. The onset of BAA can also lead to heightened anxiety, sadness, and a growing sense of emotional disconnect from the act of breastfeeding itself. These feelings may further compound the psychological burden, potentially affecting maternal confidence and well-being [32]. One study found that more than half (52.4%) of participants reported that BAA had caused them to end breastfeeding sessions before their child was ready to stop feeding (n = 210) [30]. Recent research suggests that BAA is a significant and under-recognized phenomenon among lactating women. A large Australian study found that 22.6% of 5511 breastfeeding women self-identified as having experienced BAA, representing the first known prevalence data for BAA [33]. Similarly, a scoping review identified another study reporting that 23% of participants experienced BAA, indicating a consistent prevalence across different samples [20]. In more targeted studies, higher rates have been observed, reflecting the experiences of individuals who already identify as having aversion. For example, Morns et al. [30] found that 76.7% of 210 participants who self-reported experiencing BAA described aversion throughout the feeding session while the child was latched. In the same study, 52.4% reported ending breastfeeding sessions prematurely due to BAA [30]. Together, these findings indicate that BAA is a relatively common experience that can have significant implications for maternal well-being and breastfeeding duration, highlighting the need for greater clinical awareness and supportive interventions.

#### 3.2.1. Symptomatology of BAA

**Figure 5:**
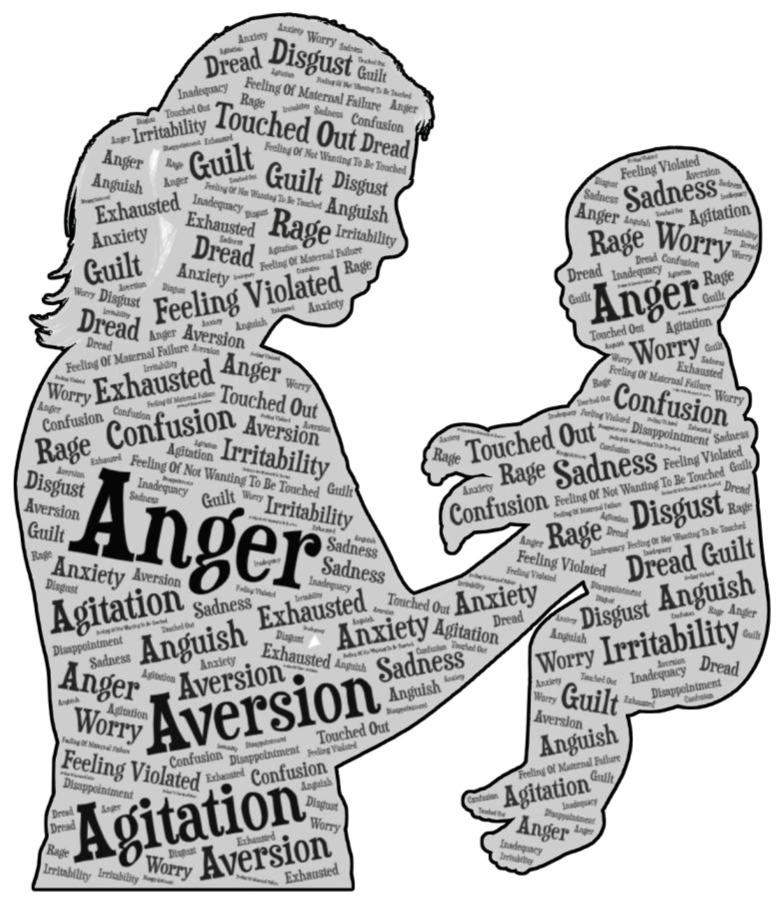
Possible symptoms of BAA/BAR.

Symptoms can be characterized by a range of intense emotional and physical sensations that start and end with the act of breastfeeding. Women frequently report a spectrum of negative emotions, including anger, irritation, sadness, and anxiety. In more severe cases, some mothers describe feelings of repulsion, disgust, or a profound sense of not wanting to be touched. A small number of participants have disclosed experiencing intrusive thoughts, including urges to harm the infant, although these are typically recognized as distressing and unwanted. These emotional responses (graphically depicted in Figure 5) are often involuntary and can lead to significant internal conflict, especially for mothers who remain committed to breastfeeding and nurturing their children [30,31].

A very common physical sensation is a skin-crawling or itchy feeling, which some describe as a neuralgia-like feeling. Other physical sensations include throat tightening and gut-wrenching sensations. Some mothers also describe an overwhelming urge to remove the suckling infant or to unlatch. Feelings of being “touched out” due to constant physical contact are also common. Affective pain descriptors such as “tiring,” “exhausting,” and “sickening” have also been used. Some women have reported feeling “violated” [20,30]. These intense experiences frequently lead to feelings of guilt, shame, and confusion, as mothers struggle to reconcile their deep desire to nurture with the visceral aversion they feel during breastfeeding. This internal dissonance can contribute to maternal distress and may ultimately impact breastfeeding continuation, especially in the absence of validation or support [34]. While sharing some similarities with D-MER, it appears that BAA/BAR is a separate problem, that might bother some of the nursing mothers. The similarities and differences are summarized in Table 2 and Table 3, respectively.

#### 3.2.2. Pathophysiology of BAA

While the pathophysiology of BAA remains unclear, several interesting observations were made. Chronic pain and physical discomfort in the form of nipple soreness associated with factors such as poor latch, infant tongue tie, and pregnancy-related sensitivity could lead to persistent irritation, if unresolved. Engaging repeatedly and knowingly in an activity that causes pain or bodily distress can contribute to negative emotional responses, including agitation, irritability, and anger. This supports the notion that physiological pain may be a contributing factor to the development or exacerbation of BAA, particularly when mothers feel unable to alleviate the underlying cause. The emotional toll of ongoing pain, especially in the context of the physical and hormonal demands of breastfeeding, may intensify the experience of aversion and further strain the breastfeeding relationship [31]. From an evolutionary perspective, BAA may function as a biological mechanism aimed at optimizing reproductive success by regulating maternal resource allocation, however pragmatic it may sound. Some researchers have suggested that BAA could serve as an evolutionary adaptation to protect parental resources and increase the likelihood of future reproduction. This theory is supported by the observation that BAA frequently arises in specific contexts—such as during pregnancy, tandem feeding, or while breastfeeding older children—situations in which the energetic and physiological demands on the mother are particularly high [20]. The aversive response may act as a signal to reduce or cease breastfeeding when it could compromise maternal health or the viability of a subsequent pregnancy—analogous to the logic behind interpregnancy interval [35]. From this point of view, BAA is not merely a maladaptive emotional state, but rather a potential regulatory mechanism that evolved to help balance investment in current offspring with the biological imperative of future reproductive opportunities. This perspective helps contextualize BAA within broader theories of parental investment and life history strategy, highlighting its possible adaptive function in response to competing reproductive demands [20,35]. Hormonal fluctuations appear to play a significant role in the onset and intensity of BAA. It was noted several times in the literature, that mothers reported that their experiences of BAA coincided with the return of their postnatal menstrual cycles. Specifically, some women noted that aversive feelings emerged around ovulation or menstruation and subsided once their period ended, suggesting a potential link between cyclical hormonal changes and the occurrence of BAA [30]. From a biological standpoint, it is possible that such experiences could represent an evolved mechanism to encourage weaning in situations where maternal nutrient levels or hormonal profiles are suboptimal for continued lactation or the body is preparing for the next pregnancy [31]. Given the vital role hormones play in regulating milk production and maternal behavior, these fluctuations may disrupt the typical neurochemical reward pathways associated with breastfeeding.

## 4. The Hormones on the Playing Field

### 4.1. Oxytocin

Oxytocin (OT) is a nonapeptide hormone composed of nine amino acids, evolutionarily derived from the ancient molecule vasotocin. In mammals, OT is primarily synthesized by magnocellular neurons located in the paraventricular nucleus and supraoptic nucleus of the hypothalamus. These neurons send axonal projections to the posterior pituitary, where oxytocin is secreted directly into the systemic circulation. In addition to this peripheral route, parvocellular neurons in the paraventricular nucleus project to various regions of the brain, releasing OT at central synapses and thus influencing a range of neurobehavioral functions. As was already mentioned, during breastfeeding, OT plays a pivotal role in the milk ejection reflex. Beyond its peripheral effects, OT also exerts central effects in the brain, promoting maternal bonding, stress reduction, and the reinforcement of nurturing behaviors—all of which are critical components of the breastfeeding relationship. That is why is OT being called “parenting hormone” [36]. Endogenous OT released during breastfeeding has been proposed to play a key role in modulating maternal mood and stress, with studies suggesting that it contributes to the anxiolytic and calming effects often observed during the postpartum period. Specifically, OT is thought to reduce anxiety and attenuate physiological stress responses in breastfeeding women, facilitating maternal-infant bonding and promoting the caregiving behaviors essential for infant survival [37]. The peripartum period is typically marked by a natural dampening of emotional and physiological reactivity, a state that has been linked to elevated central OT levels. This neurohormonal environment supports maternal behaviors and emotional regulation. In contrast, lower OT levels in postpartum women have been associated with increased rates of depression, suggesting that disruptions in the OT system may contribute to mood disorders during this vulnerable period [38]. The potential of OT’s anxiolytic effects are thought to occur via its action on brain structures involved in emotional regulation, including the amygdala, hypothalamus, and substantia nigra [39]. In the substantia nigra, oxytocin is believed to promote a quiescent, motionless posture, which may facilitate nursing by allowing the mother to remain still while the infant feeds. This neural mechanism supports the broader behavioral effects of oxytocin in encouraging calm, nurturing states that are essential for effective breastfeeding [38]. Disruptions in these regulatory pathways—whether due to hormonal fluctuations, stress, or neurological sensitivity—may help explain the emergence D-MER or BAA in some women, particularly when oxytocin’s typical calming effects are diminished or overridden. Studies involving OT gene knockout mice provide compelling evidence for oxytocin’s broad role in social behavior. Female OT knockout mice, particularly those that are nulliparous, show deficient maternal behaviors, including poor pup retrieval and nursing, pointing to oxytocin’s critical role in initiating and sustaining caregiving. In semi-natural environments, female OT-deficient mice display exaggerated aggression and enhanced anxiety-like behaviors, reflecting oxytocin’s typical role in modulating emotional responses and social tolerance [40]. OT exerts its effects via the Gq-protein coupled oxytocin receptor (OXTR), which is expressed peripherally in the mammary glands and uterus, as well as centrally in the brain, where its distribution follows a distinct spatiotemporal pattern across the lifespan. In humans, central OXTR expression begins to increase prenatally, reaching its peak during early childhood. In adult females, the cerebellar cortex demonstrates the most prominent upregulation of OXTR expression. Notably, several psychiatric, cognitive, and physiological traits, including schizophrenia, general cognitive ability, IQ, reproductive function, osteoporosis, and BMI—have been associated with the enrichment of genes showing the strongest top 100 correlations with the spatiotemporal expression profile of OXTR [41]. Mothers of infants exhibited higher expression of the OXTR compared to women without infants, indicating a potential role of oxytocin signaling in facilitating nurturing behavior. However, this upregulation of OXTR expression was attenuated in mothers with a history of early life trauma, such as childhood sexual abuse or parental loss, particularly among those with limited prior maternal experience. These findings suggest that adverse early experiences may disrupt the typical postpartum increase in OXTR expression. Furthermore, individual variability in OXTR expression—potentially influenced by epigenetic modifications—may underlie differential sensitivity to social support in the context of maternal stress [42]. Studies on oxytocin receptor knockout mice provide further insight into the essential role of oxytocin signaling in regulating social behavior, emotional reactivity, and maternal care. These models demonstrate the broad behavioral and physiological consequences of disrupting oxytocin receptor pathways, offering a neurobiological framework for interpreting aversive responses during lactation. Mice lacking oxytocin receptors exhibit pervasive social impairments, despite normal parturition. In conditional OXTR knockout females, maternal behaviors (such as nursing and grooming) appear behaviorally intact, yet these animals exhibit increased pup mortality, suggesting that while basic caregiving behaviors may persist, subtle deficits in emotional attunement or physiological regulation may compromise offspring survival [40]. Lifelong OT receptor knockout mice exhibit elevated aggression, and although post-weaning knockouts also show heightened aggression, the effect is less pronounced, highlighting a critical developmental window during which oxytocin receptor signaling shapes social-emotional behavior. Mice heterozygous for the OT receptor gene (+/−) display impaired social behavior but do not show increased aggression or cognitive inflexibility. This suggests a haploinsufficiency effect, where even partial loss of receptor function selectively impairs social affiliative behavior without broadly disrupting other functions, reinforcing the receptor’s specificity in social modulation [40]. Increased aggressive behavior has been noted in mice with lifelong versus post-weaning knockout of the oxytocin receptor [43]. The expression of the OXTR is subject to dynamic regulation throughout the lifespan, beginning with a prenatal increase and reaching its peak during early childhood. Alterations in OXTR have been implicated in several neuropsychiatric disorders, including autism spectrum disorder, depression, schizophrenia, and obsessive–compulsive disorder, although cross-population findings remain inconsistent. Several polymorphisms within the OXTR gene, notably rs2254298, rs53576, and rs1042778, have been associated with individual differences in maternal affection, sensitivity, and caregiving behavior. Hypermethylation of the OXTR gene has been correlated with perinatal depression, suggesting potential disruptions in maternal responsiveness and bonding [44]. Experimental evidence has demonstrated that blocking oxytocin signaling within key brain regions involved in emotional regulation can significantly alter maternal behavior. Specifically, the infusion of an oxytocin antagonist into the central nucleus of the amygdala in rats has been shown to increase maternal aggressive behavior [45]. Research on exogenous OT administration further underscores its role as a modulator of aggression and social behavior. In rodent models, the administration of exogenous OT—particularly via central or intranasal routes—has been shown to reduce aggressive behavior, especially in male rats. For instance, rats treated with oxytocin were significantly less aggressive towards intruders [46]. Across multiple studies, an inverse relationship has been observed between oxytocin levels and aggression: lower OT concentrations are often associated with higher levels of aggression, while elevated OT, particularly in the periphery, has been significantly linked to reduced aggression. Interestingly, the effect of exogenous OT appears to be sex-dependent. While intranasal oxytocin decreased aggression in males, at least one study reported no significant effect on female aggression, suggesting that sex-specific neuroendocrine mechanisms may influence oxytocin’s behavioral outcomes [46]. Moreover, under specific circumstances, elevated OT levels are linked to paradoxical effect on aggression. In situations related to the offspring protection, maternal aggression heightens with higher OT levels [46]. Currently, there is a lack of new data regarding the interplay between the OT and opioid systems (OS), despite the well-established role of the OS in both analgesia and a range of social behaviors, including trust, reward, impulsivity, and mating. It has long been recognized that oxytocin-producing neurons express functional opioid receptors on their terminals, which may modulate OT release [47]. Endogenous opioid peptides are known to suppress the firing rates of OT neurons [48], potentially serving as a mechanism to prevent premature oxytocin release during late pregnancy [49]. In lactating rats, comparable OT levels and similar mammary ejection reflex (MER) activity in both morphine-treated and control animals suggest the development of tolerance to the initial inhibitory effects of exogenous opioids. Nonetheless, administration of the opioid receptor antagonist naloxone significantly increases OT levels in both groups, though to varying degrees [50]. Additionally, local opioid withdrawal has been shown to upregulate oxytocin gene expression in hypothalamic magnocellular neurosecretory cells [51]. Interestingly, OT itself exerts analgesic effects, which can be attenuated by opioid receptor antagonism [52]. Its anxiolytic properties are modulated by endogenous opioids in a receptor-specific manner—either enhanced or suppressed depending on whether μ- or κ-opioid receptors are involved [53]. Although the mechanisms underlying oxytocin-opioid system interactions remain incompletely understood, recent findings from Japanese researchers have provided valuable insights. They demonstrated that oxytocin can act as a positive allosteric modulator of both μ- and κ-opioid receptors, suggesting its potential as an analgesic agent [52,54], and also found structural properties of OT, which are crucial for allosteric modulation of opioid receptor [55]. Taken together, these findings indicate that the interaction between the oxytocinergic and opioidergic systems significantly shapes social behavior, producing effects beyond those attributable to either neuromodulator alone. In the context of maternal behavior, these interactions support the hypothesis that OT functions as a neuromodulator facilitating maternal calmness and suppressing excessive defensive responses, thereby promoting nurturing and protective caregiving. In relation to BAA, such evidence aligns with the notion that reduced central OT activity—whether due to hormonal fluctuations, stress, or receptor-level dysregulation—may contribute to heightened irritability, agitation, or aggression during lactation.

### 4.2. Prolactin

Prolactin (PRL) is a highly versatile polypeptide hormone with more than 300 identified physiological functions. It was originally named for its role in stimulating lactation and promoting mammary gland development. Prolactin is primarily produced and secreted by lactotroph cells located in the anterior pituitary gland, where these cells account for approximately 15–25% of the total cell population in a healthy human pituitary. Its secretion is largely under inhibitory regulation by dopamine. Although the anterior pituitary is the principal source, prolactin is also synthesized and secreted by various extrapituitary tissues, including the decidua, mammary glands, ovaries, prostate, testes, vascular endothelium, lymph nodes, skin, adipose tissue, inner ear cochlea, and immune cells [56]. The broad range of prolactin’s biological actions is mediated by its transmembrane receptor (PRL-R), a member of the haematopoietic type 1 cytokine receptor superfamily. Multiple PRL-R isoforms arise through alternative mRNA splicing, characterized by identical extracellular domains but variable intracellular domains. PRL-Rs are widely distributed throughout numerous tissues in the body, including prominent expression within the brain [57]. Among its numerous physiological roles, prolactin is recognized as an adaptive hormone contributing to the stress response and is critically involved in reproductive and maternal behaviors. It is indispensable for the initiation and maintenance of maternal care across mammalian species. Experimental studies in rats have shown that exogenous administration of prolactin to virgin females induces maternal behaviors and significantly reduces the latency to their onset. Conversely, pharmacological inhibition of prolactin secretion using bromocriptine results in delayed onset of maternal behaviors and impaired parenting abilities [58]. The medial preoptic area (MPOA) of the hypothalamus constitutes a key neural substrate mediating PRL-dependent maternal behaviors. Experimental studies have demonstrated that the infusion of PRL or placental lactogen into the MPOA is sufficient to induce maternal behavior in non-pregnant rats. Furthermore, conditional deletion of the PRL-R within the MPOA in mice results in a complete disruption of postpartum maternal behavior. PRL also promotes neurogenesis within the subventricular zone during early pregnancy, a process critical for establishing appropriate postpartum behavioral responses, particularly in stressful environments. A decrease in PRL levels during this critical window impairs neurogenesis and is associated with deficits in offspring retrieval and increased anxiety-like behavior in the postpartum period [58,59]. PRL signaling plays a pivotal role in the regulation of offspring-directed behaviors, including pup retrieval, licking, nursing, and grooming, as well as offspring-associated behaviors such as anxiety modulation, nest building, and maternal aggression [60]. Maternal exposure to prolactin, potentially mediated through lactational transfer, may contribute to the programming of offspring neuronal systems involved in the regulation of maternal behavior in later life. Experimental reductions in prolactin concentrations within rat milk have been associated with impaired maternal behaviors in adult female offspring [59]. PRL contributes to the regulation of the stress response predominantly through the inhibition of hypothalamic–pituitary–adrenal axis activity, as was already mentioned. It attenuates emotional, hormonal, and neuronal reactivity to diverse stressors [56]. During pregnancy and lactation, the reactivity of the hypothalamic–pituitary–adrenal axis is markedly reduced, a critical adaptation that serves to protect both the mother and offspring from the detrimental effects of stress-related physiological responses [59]. PRL possesses anxiolytic properties, as evidenced by findings that chronic prolactin administration reduces adrenocorticotropic hormone (ACTH) and corticosterone secretion following exposure to stress, and diminishes neuronal activation in the paraventricular nucleus—a principal center for stress integration [59]. Additionally, the downregulation of PRL-R expression in the brain has been demonstrated to result in increased anxiety-like behaviors and elevated ACTH levels in lactating rodent models [59]. Offspring of dams subjected to chronic social stress have been shown to exhibit impaired social behaviors, which are accompanied by reduced basal plasma PRL concentrations [58]. Prolactin has been implicated in the promotion of resilience within a chronic mild stress model in rats. Resilient animals exhibited elevated plasma prolactin concentrations and increased PRL-R mRNA expression in the choroid plexus compared to their vulnerable counterparts. These findings suggest a potential role for prolactin in the regulation of stress responses at the level of the hippocampus [56]. Experimental studies in rats have demonstrated that the artificial induction of hyperprolactinemia can produce antidepressant-like effects [58]. In humans, however, the relationship between PRL levels and depressive symptoms appears to be complex. While certain individuals with hyperprolactinemia exhibit features of depression, the overall prevalence of depressive symptoms does not consistently differ from that of the general population [56,58]. Furthermore, the administration of prolactin during early postnatal development in rats has been shown to impair neurogenesis and promote a depressive-like phenotype in adulthood, indicating that prolactin’s effects on mood may be age-dependent [61]. Clinically, decreased maternal serum PRL levels have been correlated with the presence of depressive symptoms during late gestation and the postpartum period, as well as with reduced social interactive behavior in neonates. It has been proposed that maternal PRL levels may influence the offspring’s early social behaviors [58]. Reduced PRL concentrations in lactating women have been associated with an increased risk of developing postpartum depression [61]. The administration of bromocriptine, a dopamine agonist that suppresses PRL secretion, during the early stages of pregnancy in rats has been shown to induce a depressive-like state and impair maternal behaviors, suggesting that PRL may play a critical role in the pathophysiology of postpartum depression [62]. Interestingly, a study from 2022 reported contradictory results, claiming that patients with postpartum depression exhibited significantly higher serum PRL concentrations compared to healthy postpartum women. Moreover, elevated PRL levels in postpartum depression patients were found to be positively correlated with scores on the Edinburgh Postnatal Depression Scale [63]. Maternal perinatal depressive symptoms have been associated with reduced maternal serum PLR levels, accompanied by elevated maternal and neonatal serum cortisol concentrations. Chronic stress or depression can contribute to dysregulation of the hypothalamic–pituitary–adrenal axis, resulting in excessive cortisol secretion and suppressed prolactin expression. Prolactin is generally regarded as an adaptive hormone involved in the modulation of the stress response. Under typical stress conditions, prolactin secretion increases, serving to attenuate hypothalamic–pituitary–adrenal axis hyperactivity. However, during the perinatal period, the presence of chronic stress or depressive symptoms may disrupt this regulatory mechanism [58]. PRL may play a role in the regulation of maternal aggression, a behavior commonly exhibited by postpartum females. Increased levels of postpartum aggression have been observed in breastfeeding women, who typically display elevated prolactin concentrations. Furthermore, women diagnosed with hyperprolactinemia have been reported to exhibit similar or higher levels of hostility compared to control groups in certain studies [64]. Elevated PRL concentrations can lead to increased production of the 16K PRL fragment, known as vasoinhibin, which exerts biological effects opposite to those of the native hormone. Vasoinhibin has been associated with the promotion of anxiety- and depression-related behaviors [56].

### 4.3. Dopamine

Dopamine (DA) is a critical monoamine neurotransmitter that plays a central role in a wide range of physiological processes within the central nervous system. It is synthesized from the amino acid tyrosine through a two-step enzymatic process, wherein tyrosine hydroxylase catalyzes the conversion of tyrosine to L-DOPA, followed by the action of DOPA decarboxylase, which converts L-DOPA to DA. Once synthesized, dopamine is transported into synaptic vesicles via the vesicular monoamine transporter 2, facilitating its storage and regulated release. Dopaminergic neurons originate from several distinct brain regions and project to various targets, forming specific dopaminergic pathways. Key production sites include substantia nigra, ventral tegmental area and hypothalamus [65]. Beyond their anatomical proximity, OT and DA actively modulate each other’s release. DA receptors are expressed on oxytocinergic neurons within the paraventricular nucleus, and DA has been shown to stimulate OT release [66]. In particular, D2 and D3 receptors appear to activate oxytocinergic neurons. Conversely, OT can enhance DA levels in several brain regions—including the medial preoptic area, amygdala, and ventral tegmental area—all of which express OT receptors. Notably, central administration of an OT antagonist has been found to reduce DA release in response to a dopamine agonist [66,67]. OT also exerts both direct and indirect effects on DA neurons. In slice recording studies, application of OT increased the firing rates of ventral tegmental area DA neurons while decreasing the firing rates of dopamine neurons in the lateral substantia nigra pars compacta. Optogenetic stimulation of OT fibers produced similar opposing effects: enhancing ventral tegmental area DA neuron activity while suppressing lateral substantia nigra pars compacta DA neuron activity. The enhancement of ventral tegmental area firing was blocked by an OT receptor antagonist, suggesting direct modulation, whereas the inhibition of substantia nigra pars compacta neurons appeared to occur indirectly through local GABAergic circuits. These dual actions highlight the nuanced role of OT in regulating the brain’s reward pathways [66]. DA is engaged in a broad array of critical physiological and behavioral functions. The ones most relevant to this review are: learning and memory (particularly those processes associated with hedonic and motivational rewards), affective and emotional states (including mood regulation), cognitive functions (including attention, executive functioning, and decision-making) and social behavior (modulating both the pleasurable and aversive aspects of social interactions and influencing affiliative behaviors) [65,68]. In the same context, dysregulation of the dopaminergic system is implicated in the pathophysiology of multiple neurological and psychiatric disorders, including: schizophrenia (associated with hyperactivity of limbic and cortical dopaminergic circuits), bipolar disorder (the dopamine hypothesis posits that faulty homeostasis between dopamine receptors and transporters contributes to manic and depressive phases), major depressive disorder (linked to deficits and dysregulation in dopaminergic signaling, particularly affecting reward processing and contributing to anhedonia) and attention-deficit/hyperactivity disorder (involving alterations in dopaminergic transmission) [69,70,71]. The interaction between OT and DA is crucial for a range of reward-related social behaviors. For example, pair bond formation in female voles depends on the simultaneous activation of OT and D2 receptors within the nucleus accumbens. Similarly, the initiation and maintenance of maternal behavior are driven by OT’s modulation of DA release in the mesocorticolimbic system, with mothers exhibiting stronger maternal behaviors showing increased OT projections to the ventral tegmental area and greater DA release in the nucleus accumbens. Even sexual behavior in male rats involves DA-driven activation of specific OT neurons, depending on the behavioral context [72]. Anhedonia is defined as a reduced interest in or diminished pleasure from stimuli that were previously experienced as rewarding. The dopaminergic system is believed to play a pivotal role in the development of hedonic deficits observed in this condition, which closely mimics the psychological state of D-MER. The dopaminergic system is critically involved in reward prediction, motivational arousal, and responsiveness to conditioned incentive stimuli. It has been proposed that dopamine is essential for the attribution of incentive salience to motivationally relevant stimuli, effectively transforming the experience of liking a reward into a goal-directed wanting. Disruptions in this dopaminergic function are commonly observed in individuals with major depressive disorder, manifesting as a diminished motivation to seek pleasurable experiences [69]. Anhedonia encompasses a range of reward-processing deficits, including impairments in the anticipation, motivation, and decision-making processes necessary for obtaining rewards. It is strongly associated with dysfunctions in the brain’s reward system, with particular emphasis on dopaminergic dysregulation [67]. Certain disorders marked by social deficits, like autism spectrum disorder and schizophrenia, display disruptions in both OT and DA signaling. Given OT’s role in social attachment, affiliation, and enhancing the salience of social cues—alongside DA’s involvement in reward and motivation during social interactions—it is likely that imbalances between these systems contribute to social impairments. For instance, research using autism mouse models has shown that OT administration can boost both social behaviors and dopaminergic activity [73].

## 5. Future Research

Future research should aim to deepen understanding of the causes, prevalence, clinical features, and effective management of D-MER and BAA. Given the current limitations in empirical data, several key areas require focused investigation. First, the underlying mechanisms need to be explored. In the case of D-MER, this involves examining the role of dopamine regulation and its interaction with prolactin and oxytocin during milk ejection. For BAA, further research is needed to determine whether its origins are physiological, psychological, or both, considering factors such as hormonal fluctuations, sensory overload, and psychosocial stressors.

Epidemiological studies are essential to estimate prevalence, identify risk factors, and clearly distinguish these conditions from each other and from related perinatal mood disorders. In parallel, the development of standardized diagnostic tools or screening questionnaires would support earlier identification and facilitate their integration into routine postpartum care, enabling professionals such as lactation consultants, midwives, and primary care providers to better recognize and address these issues.

It is also important to examine the impact of D-MER and BAA on breastfeeding duration, maternal mental health, and the mother-infant relationship. Clinical trials will be needed to assess the effectiveness of both pharmacological and non-pharmacological interventions, including cognitive-behavioral therapy.

## 6. Conclusions

The act of breastfeeding is hormonally complex, orchestrated by the finely tuned interactions of oxytocin, prolactin, and dopamine. These systems not only support lactation but also modulate maternal emotional states, bonding behaviors, and stress regulation. However, in some women, dysregulation within these pathways can give rise to distinct conditions such as D-MER and BAA, profoundly impacting the breastfeeding experience. Oxytocin, often celebrated as the “parenting hormone,” typically reduces anxiety, facilitates bonding, and promotes maternal calmness. Yet, paradoxically, under conditions of previous trauma, chronic stress, or individual neurobiological vulnerability, oxytocin may trigger heightened stress responses instead of promoting relaxation. This altered oxytocin activity may contribute to the dysphoric sensations characteristic of D-MER and the intense irritability observed in BAA. Evidence from oxytocin knockout models and intranasal oxytocin administration further supports oxytocin’s dual potential to modulate both affiliative and defensive behaviors, depending on context and receptor sensitivity. Similarly, prolactin, traditionally associated with maternal behaviors and stress attenuation, displays a complex relationship with emotional regulation. While elevated prolactin generally supports nurturing behaviors and anxiolysis, chronic stress, hyperprolactinemia, or disruptions in prolactin signaling can paradoxically promote anxiety, depressive symptoms, and impaired maternal care. Variations in prolactin exposure during critical developmental windows may also impact the social and emotional development of offspring, highlighting prolactin’s broader role in intergenerational mental health. Dopamine plays an equally critical role, particularly through its regulation of motivational salience and reward processing. Disruptions in dopaminergic tone may underlie the anhedonia, irritability, and emotional blunting reported in D-MER and postpartum mood disorders. The observation that dopamine stabilizers can alleviate D-MER symptoms lends credence to the dopamine hypothesis of lactational dysphoria. Moreover, the parallels between dopaminergic dysfunction in major depressive disorder and breastfeeding-related emotional aversion suggest a shared neurobiological substrate. Importantly, the relationship between these neuroendocrine systems is not linear but interactive. Dopamine, prolactin, and oxytocin influence each other’s secretion and receptor sensitivity, creating a dynamic feedback loop that can either stabilize or destabilize emotional homeostasis during lactation. Chronic stress, perinatal adversity, and hormonal fluctuations may tip this balance toward dysregulation, increasing the risk of aversive breastfeeding experiences. Clinically, these insights highlight the need for increased awareness and validation of maternal emotional experiences during breastfeeding. Many women suffering from D-MER or BAA experience profound guilt, shame, and confusion, often exacerbated by the societal idealization of breastfeeding. Routine screening for breastfeeding-related emotional disturbances, alongside education about D-MER and BAA, could empower mothers to seek appropriate support and make informed decisions about their feeding journey. Future research should aim to further elucidate the neuroendocrine mechanisms underlying D-MER and BAA, investigate potential predictive biomarkers, and explore therapeutic strategies, including targeted hormonal modulation, psychological interventions, and supportive care models. Understanding that negative emotional responses during breastfeeding are biologically rooted rather than reflective of maternal failure is essential for advancing compassionate, evidence-based postpartum care.

## Figures and Tables

**Table 1:** The most common reasons for premature breastfeeding cessation.

Reasons for Premature Breastfeeding Cessation	
perception of inadequate milk supply	late lactogenesis
perception of insufficient nutrition	return to work
lack of support from family members	unrealistic expectations
concerns about the safety of medication taken	maternal obesity
uncoordinated suck and swallow reflex	smoking
maternal mental health problems	complicated childbirth

**Table 2:** Similarities between D-MER and BAA/BAR.

Aspect	D-MER and BAA/BAR Similarities
Associated with breastfeeding	both occur in breastfeeding individuals
Emotional/psychological symptoms	both involve distressing emotional experiences
Trigger	both are triggered or worsened by nursing or milk release
Impact	can lead to early weaning or reduced breastfeeding satisfaction
Recognition	under-recognized and often misunderstood by healthcare providers; both require emotional support and sometimes professional care

**Table 3:** Differences between D-MER and BAA/BAR.

**Aspect**	**D-MER**	**BAA/BAR**
Definition	a neurological condition causing brief dysphoria during let-down	psychological aversion or agitation toward nursing
Onset timing	occurs just before or during milk ejection (let-down reflex)	can occur anytime during nursing, especially with older infants
Duration of episode	lasts 30 s to 2 min	persists as long as nursing continues
Primary emotions	sudden wave of sadness, dread, anxiety	anger, irritability, skin-crawling sensation
Hormonal involvement	linked to hormonal changes during let-down	not clearly linked to hormonal shifts
Typical onset period	often appears in early postpartum period	can develop later, especially during toddler nursing or tandem feeding
Resolution	often resolves over time	may persist unless feeding patterns are changed
Research status	some emerging scientific understanding	largely anecdotal, with limited formal study

## Abbreviations

The following abbreviations are used in this manuscript:

D-MER	Dysphoric Milk Ejection Reflex
BAA	Breastfeeding Aversion/Agitation
PPD	postpartum depression
PTSD	post-traumatic stress disorder
OT	oxytocin
OXTR	oxytocin receptor
PRL	prolactin
PRL-R	prolactin receptor
MPOA	medial preoptic area
ACTH	adrenocorticotropic hormone
DA	dopamine

## References

[1] Eglash A., Montgomery A., Wood J. (2008). Breastfeeding. Disease-A-Month.

[2] Yi D., Kim S. (2021). Human Breast Milk Composition and Function in Human Health: From Nutritional Components to Microbiome and MicroRNAs. Nutrients.

[3] Miles E.A., Childs C.E., Calder P.C. (2021). Long-Chain Polyunsaturated Fatty Acids (LCPUFAs) and the Developing Immune System: A Narrative Review. Nutrients.

[4] Modak A., Ronghe V., Gomase K.P. (2023). The Psychological Benefits of Breastfeeding: Fostering Maternal Well-Being and Child Development. Cureus.

[5] Brockway M.M., Daniel A.I., Reyes S.M., Granger M., McDermid J.M., Chan D., Refvik R., Sidhu K.K., Musse S., Patel P.P. (2024). Human Milk Macronutrients and Child Growth and Body Composition in the First Two Years: A Systematic Review. Adv. Nutr..

[6] Goldman A.S., Chheda S. (2021). The Immune System in Human Milk: A Historic Perspective. Ann. Nutr. Metab..

[7] Sayres S., Visentin L. (2018). Breastfeeding: Uncovering Barriers and Offering Solutions. Curr. Opin. Pediatr..

[8] Westerfield K.L., Koenig K., Oh R. (2018). Breastfeeding: Common Questions and Answers. Am. Fam. Physician.

[9] Billings H., Horsman J., Soltani H., Spencer R.L. (2024). Breastfeeding Experiences of Women with Perinatal Mental Health Problems: A Systematic Review and Thematic Synthesis. BMC Pregnancy Childbirth.

[10] Xia M., Luo J., Wang J., Liang Y. (2022). Association between Breastfeeding and Postpartum Depression: A Meta-Analysis. J. Affect. Disord..

[11] Mezzacappa E.S. (2004). Breastfeeding and Maternal Stress Response and Health. Nutr. Rev..

[12] Bugaeva P., Arkusha I., Bikaev R., Kamenskiy I., Pokrovskaya A., El-Taravi Y., Caso V., Avedisova A., Chu D.K., Genuneit J. (2023). Association of Breastfeeding with Mental Disorders in Mother and Child: A Systematic Review and Meta-Analysis. BMC Med..

[13] Coo S., García M.I., Prieto F. (2024). Quality of Mother-Infant Interaction, Breastfeeding, and Perinatal Mental Health. Infant Behav. Dev..

[14] Cohen S.S., Alexander D.D., Krebs N.F., Young B.E., Cabana M.D., Erdmann P., Hays N.P., Bezold C.P., Levin-Sparenberg E., Turini M. (2018). Factors Associated with Breastfeeding Initiation and Continuation: A Meta-Analysis. J. Pediatr..

[15] Dias C.C., Figueiredo B. (2015). Breastfeeding and Depression: A Systematic Review of the Literature. J. Affect. Disord..

[16] Stuebe A.M., Meltzer-Brody S., Propper C., Pearson B., Beiler P., Elam M., Walker C., Mills-Koonce R., Grewen K. (2019). The Mood, Mother, and Infant Study: Associations Between Maternal Mood in Pregnancy and Breastfeeding Outcome. Breastfeed. Med..

[17] Henshaw E.J. (2023). Breastfeeding and Postpartum Depression: A Review of Relationships and Potential Mechanisms. Curr. Psychiatry Rep..

[18] Frawley T., McGuinness D. (2023). Dysphoric Milk Ejection Reflex (D-MER) and Its Implications for Mental Health Nursing. Int. J. Ment. Health Nurs..

[19] Heise A.M., Wiessinger D. (2011). Dysphoric Milk Ejection Reflex: A Case Report. Int. Breastfeed. J..

[20] Middleton C., Lee E., McFadden A. (2025). Negative Emotional Experiences of Breastfeeding and the Milk Ejection Reflex: A Scoping Review. Int. Breastfeed. J..

[21] Ureño T.L., Berry-Cabán C.S., Adams A., Buchheit T.L., Hopkinson S.G. (2019). Dysphoric Milk Ejection Reflex: A Descriptive Study. Breastfeed. Med..

[22] Ahmed M., Mahmud A., Mughal S., Shah H.H. (2024). Dysphoric Milk Ejection Reflex—Call for Future Trials. Arch. Gynecol. Obstet..

[23] Solmonovich R.L., Kouba I., Bailey C., Andria W., Demertzis K., Blitz M.J., Muscat J. (2025). Incidence and Awareness of Dysphoric Milk Ejection Reflex (DMER). J. Perinat. Med..

[24] Cappenberg R., Garcia J.G., Liolios I., Happle C., Zychlinsky Scharff A. (2025). Dysphoric Milk Ejection Reflex: Prevalence, Persistence, and Implications. Eur. J. Obstet. Gynecol. Reprod. Biol..

[25] Moriyama Y., Nakao Y., Yamamoto N., Oki T. (2024). Dysphoric Milk Ejection Reflex among Japanese Mothers: A Self-Administered Survey. Int. Breastfeed. J..

[26] Watkinson M., Murray C., Simpson J. (2016). Maternal Experiences of Embodied Emotional Sensations during Breast Feeding: An Interpretative Phenomenological Analysis. Midwifery.

[27] Deif R., Burch E.M., Azar J., Yonis N., Abou Gabal M., El Kramani N., DakhlAllah D. (2021). Dysphoric Milk Ejection Reflex: The Psychoneurobiology of the Breastfeeding Experience. Front. Glob. Womens Health.

[28] Uvnas-Moberg K., Kendall-Tackett K. (2018). The Mystery of D-MER: What Can Hormonal Research Tell Us About Dysphoric Milk-Ejection Reflex?. Clin. Lact..

[29] Ureño T.L., Buchheit T.L., Hopkinson S.G., Berry-Cabán C.S. (2018). Dysphoric Milk Ejection Reflex: A Case Series. Breastfeed. Med..

[30] Morns M.A., Steel A.E., McIntyre E., Burns E. (2023). Breastfeeding Aversion Response (BAR): A Descriptive Study. J. Midwifery Women’s Health.

[31] Yate Z. (2017). A Qualitative Study on Negative Emotions Triggered by Breastfeeding; Describing the Phenomenon of Breastfeeding/Nursing Aversion and Agitation in Breastfeeding Mothers. Iran. J. Nurs. Midwifery Res..

[32] Morns M.A., Steel A.E., Burns E., McIntyre E. (2021). Women Who Experience Feelings of Aversion While Breastfeeding: A Meta-Ethnographic Review. Women Birth.

[33] Morns M.A., Burns E., McIntyre E., Steel A.E. (2023). The Prevalence of Breastfeeding Aversion Response in Australia: A National Cross-sectional Survey. Matern. Child Nutr..

[34] Leeming D., Marshall J., Hinsliff S. (2022). Self-conscious Emotions and Breastfeeding Support: A Focused Synthesis of UK Qualitative Research. Matern. Child Nutr..

[35] Mühlrad H., Björkegren E., Haraldson P., Bohm-Starke N., Kopp Kallner H., Brismar Wendel S. (2022). Interpregnancy Interval and Maternal and Neonatal Morbidity: A Nationwide Cohort Study. Sci. Rep..

[36] Feldman R., Bakermans-Kranenburg M.J. (2017). Oxytocin: A Parenting Hormone. Curr. Opin. Psychol..

[37] Whitley J., Wouk K., Bauer A.E., Grewen K., Gottfredson N.C., Meltzer-Brody S., Propper C., Mills-Koonce R., Pearson B., Stuebe A. (2020). Oxytocin during Breastfeeding and Maternal Mood Symptoms. Psychoneuroendocrinology.

[38] Jurek B., Neumann I.D. (2018). The Oxytocin Receptor: From Intracellular Signaling to Behavior. Physiol. Rev..

[39] Florea T., Palimariciuc M., Cristofor A.C., Dobrin I., Chiriță R., Bîrsan M., Dobrin R.P., Pădurariu M. (2022). Oxytocin: Narrative Expert Review of Current Perspectives on the Relationship with Other Neurotransmitters and the Impact on the Main Psychiatric Disorders. Medicina.

[40] Inada K. (2024). Neurobiological Mechanisms Underlying Oxytocin-Mediated Parental Behavior in Rodents. Neurosci. Res..

[41] Rokicki J., Kaufmann T., De Lange A.-M.G., Van Der Meer D., Bahrami S., Sartorius A.M., Haukvik U.K., Steen N.E., Schwarz E., Stein D.J. (2022). Oxytocin Receptor Expression Patterns in the Human Brain across Development. Neuropsychopharmacology.

[42] Light A.E., Holt-Lunstad J., Porter C.L., Light K.C. (2019). Early Life Trauma: An Exploratory Study of Effects on OXTR and NR3C1 Gene Expression and Nurturing Self-Efficacy in Mothers of Infants. Int. J. Psychophysiol..

[43] Dhakar M.B., Rich M.E., Reno E.L., Lee H.-J., Caldwell H.K. (2012). Heightened Aggressive Behavior in Mice with Lifelong versus Postweaning Knockout of the Oxytocin Receptor. Horm. Behav..

[44] Pierzynowska K., Gaffke L., Żabińska M., Cyske Z., Rintz E., Wiśniewska K., Podlacha M., Węgrzyn G. (2023). Roles of the Oxytocin Receptor (OXTR) in Human Diseases. Int. J. Mol. Sci..

[45] Lubin D.A., Elliot J.C., Black M.C., Johns J.M. (2003). An Oxytocin Antagonist Infused into the Central Nucleus of the Amygdala Increases Maternal Aggressive Behavior. Behav. Neurosci..

[46] Zhou H., Zhu R., Xia Y., Zhang X., Wang Z., Lorimer G.H., Ghiladi R.A., Bayram H., Wang J. (2024). Neuropeptides Affecting Social Behavior in Mammals: Oxytocin. Peptides.

[47] Zhao B.-G., Chapman C., Bicknell R.J. (1988). Functional κ-Opioid Receptors on Oxytocin and Vasopressin Nerve Terminals Isolated from the Rat Neurohypophysis. Brain Res..

[48] Brown C.H., Stern J.E., Jackson K.L.M., Bull P.M., Leng G., Russell J.A. (2005). Morphine Withdrawal Increases Intrinsic Excitability of Oxytocin Neurons in Morphine-dependent Rats. Eur. J. Neurosci..

[49] Douglas A.J., Johnstone L.E., Neumann I., Leng G., Russell J.A. (1994). Oxytocin Neurones in the Supraoptic Nucleus (SON) Are Inhibited by Endogenous Opioids in Late Pregnant Rats. Gene Ther..

[50] Bicknell R.J., Leng G., Lincoln D.W., Russell J.A. (1988). Naloxone Excites Oxytocin Neurones in the Supraoptic Nucleus of Lactating Rats after Chronic Morphine Treatment. J. Physiol..

[51] Johnstone L.E., Brown C.H., Meeren H.K.M., Vuijst C.L., Brooks P.J., Leng G., Russell J.A. (2000). Local Morphine Withdrawal Increases c *-Fos* Gene, Fos Protein, and Oxytocin Gene Expression in Hypothalamic Magnocellular Neurosecretory Cells. J. Neurosci..

[52] Meguro Y., Miyano K., Hirayama S., Yoshida Y., Ishibashi N., Ogino T., Fujii Y., Manabe S., Eto M., Nonaka M. (2018). Neuropeptide Oxytocin Enhances μ Opioid Receptor Signaling as a Positive Allosteric Modulator. J. Pharmacol. Sci..

[53] Nisbett K.E., Vendruscolo L.F., Koob G.F. (2024). Μ-Opioid Receptor Antagonism Facilitates the Anxiolytic-like Effect of Oxytocin in Mice. Transl. Psychiatry.

[54] Miyano K., Yoshida Y., Hirayama S., Takahashi H., Ono H., Meguro Y., Manabe S., Komatsu A., Nonaka M., Mizuguchi T. (2021). Oxytocin Is a Positive Allosteric Modulator of κ-Opioid Receptors but Not δ-Opioid Receptors in the G Protein Signaling Pathway. Cells.

[55] Mizuguchi T., Miyano K., Yamauchi R., Yoshida Y., Takahashi H., Yamazaki A., Ono H., Inagaki M., Nonaka M., Uezono Y. (2023). The First Structure-Activity Relationship Study of Oxytocin as a Positive Allosteric Modulator for the µ Opioid Receptor. Peptides.

[56] Torner L. (2016). Actions of Prolactin in the Brain: From Physiological Adaptations to Stress and Neurogenesis to Psychopathology. Front. Endocrinol..

[57] Szewczyk A.K., Ulutas S., Aktürk T., Al-Hassany L., Börner C., Cernigliaro F., Kodounis M., Lo Cascio S., Mikolajek D., Onan D. (2023). Prolactin and Oxytocin: Potential Targets for Migraine Treatment. J. Headache Pain.

[58] Zhang H., Su Q., Yao D., Wang S., Dang S., Ding D., Zhu Z., Shao S., Li H. (2017). Prolactin, a Potential Mediator of Reduced Social Interactive Behavior in Newborn Infants Following Maternal Perinatal Depressive Symptoms. J. Affect. Disord..

[59] Georgescu T., Swart J.M., Grattan D.R., Brown R.S.E. (2021). The Prolactin Family of Hormones as Regulators of Maternal Mood and Behavior. Front. Glob. Womens Health.

[60] Bernard V., Young J., Binart N. (2019). Prolactin—A Pleiotropic Factor in Health and Disease. Nat. Rev. Endocrinol..

[61] Tost M., Monreal J.A., Armario A., Barbero J.D., Cobo J., García-Rizo C., Bioque M., Usall J., Huerta-Ramos E., PNECAT Group (2020). Targeting Hormones for Improving Cognition in Major Mood Disorders and Schizophrenia: Thyroid Hormones and Prolactin. Clin. Drug Investig..

[62] Dessì A., Pianese G., Mureddu P., Fanos V., Bosco A. (2024). From Breastfeeding to Support in Mothers’ Feeding Choices: A Key Role in the Prevention of Postpartum Depression?. Nutrients.

[63] Cheng B., Hu X., Roberts N., Zhao Y., Xu X., Zhou Y., Tan X., Chen S., Meng Y., Wang S. (2022). Prolactin Mediates the Relationship between Regional Gray Matter Volume and Postpartum Depression Symptoms. J. Affect. Disord..

[64] Georgescu T., Khant Aung Z., Grattan D.R., Brown R.S.E. (2022). Prolactin-Mediated Restraint of Maternal Aggression in Lactation. Proc. Natl. Acad. Sci. USA.

[65] Speranza L., Di Porzio U., Viggiano D., De Donato A., Volpicelli F. (2021). Dopamine: The Neuromodulator of Long-Term Synaptic Plasticity, Reward and Movement Control. Cells.

[66] Rappeneau V., Castillo Díaz F. (2024). Convergence of Oxytocin and Dopamine Signalling in Neuronal Circuits: Insights into the Neurobiology of Social Interactions across Species. Neurosci. Biobehav. Rev..

[67] Petersson M., Uvnäs-Moberg K. (2024). Interactions of Oxytocin and Dopamine—Effects on Behavior in Health and Disease. Biomedicines.

[68] Klein M.O., Battagello D.S., Cardoso A.R., Hauser D.N., Bittencourt J.C., Correa R.G. (2019). Dopamine: Functions, Signaling, and Association with Neurological Diseases. Cell Mol. Neurobiol..

[69] Belujon P., Grace A.A. (2017). Dopamine System Dysregulation in Major Depressive Disorders. Int. J. Neuropsychopharmacol..

[70] Gogarnoiu E.S., Vogt C.D., Sanchez J., Bonifazi A., Saab E., Shaik A.B., Soler-Cedeño O., Bi G.-H., Klein B., Xi Z.-X. (2023). Dopamine D_3_ /D_2_ Receptor Ligands Based on Cariprazine for the Treatment of Psychostimulant Use Disorders That May Be Dual Diagnosed with Affective Disorders. J. Med. Chem..

[71] Delva N.C., Stanwood G.D. (2021). Dysregulation of Brain Dopamine Systems in Major Depressive Disorder. Exp. Biol. Med..

[72] Grieb Z.A., Lonstein J.S. (2022). Oxytocin Interactions with Central Dopamine and Serotonin Systems Regulate Different Components of Motherhood. Phil. Trans. R. Soc. B.

[73] Baskerville T.A., Douglas A.J. (2010). Dopamine and Oxytocin Interactions Underlying Behaviors: Potential Contributions to Behavioral Disorders. CNS Neurosci. Ther..

